# Multihost/Multivector Community Network: Disentangling Sandfly Species and Host Interactions in Avian Habitats

**DOI:** 10.1155/tbed/9259030

**Published:** 2024-12-17

**Authors:** J. Veiga, F. Collantes, L. M. Hernández-Triana, S. W. J. Prosser, F. Valera

**Affiliations:** ^1^Department of Parasitology, University of Granada, Granada, Spain; ^2^Department of Functional and Evolutionary Ecology, Experimental Station of Arid Zones (EEZA-CSIC), Ctra. de Sacramento s/n, La Cañada de San Urbano, Almería, Spain; ^3^Department of Zoology and Physical Anthropology, Faculty of Biology, University of Murcia, Murcia, Spain; ^4^Vector-Borne Diseases Research Group, Virology Department, Animal and Plant Health Agency, New Haw, Addlestone, Surrey, UK; ^5^Centre for Biodiversity Genomics, University of Guelph, Ontario, Canada

**Keywords:** anthropophilia, arid areas, blood meal, endophagy, phlebotomine, vector-borne

## Abstract

Ascertaining the feeding behavior of vectors is a key for understanding epidemiology of the infections they transmit. For some host–vector–parasite systems, this information is biased towards human and peridomestic habitats, frequently underestimating the likely role of wildlife. In addition, studies on vector interactions often focus on a one-to-one host–vector relationship, even though it is crucial to analyze how multiple vector species interact with multiple hosts. These biases particularly affect our knowledge of sandflies, the main vector of *Leishmania* spp. and various phleboviruses, that are rarely explored in non-peridomestic habitats and in the context of multiple interactions with various hosts. To reveal the multihost/multivector network involving phlebotomine sandflies in a semiarid and poorly populated area of Spain, we sampled the sandfly community close to avian nests by means of two trapping methods (Centers for Disease Control (CDC) and sticky traps) during 3 years and identified the blood-meal source of all engorged sandflies. We detected six phlebotomine species with *Phlebotomus perniciosus*, *P. papatasi*, and *Sergentomyia minuta* being the most abundant ones. We identified 13 blood source species, with humans being the most common one, followed by *Coracias garrulus* (European roller) and *Sus scrofa* (likely wild boar). Five of the six sandfly species fed largely on wild mammals, although, three also fed on wild birds. *Phlebotomus sergenti* only fed on birds based on this analysis. *Phlebotomus papatasi* and *P. sergenti* were common visitors of bird nests suggesting an endophagic behavior. A network analysis showed a highly-connected and poorly-specialized network wherein sandflies shared most of the blood source and showed an opportunistic feeding behavior with marked anthropophilia. Our results obtained close to avian nests show that sandfly populations are maintained by various wild animals, which will greatly complicate the management and control of the pathogens they transmit to humans and domestic animals.

## 1. Introduction

The feeding behavior of blood-sucking insects determines the transmission, distribution, host range, and evolution of blood parasites in the wild. Females of blood-sucking vectors usually have several blood sources [1], bringing different parasites into contact with different hosts. Therefore, vector–host preference is an important determinant of the incidence of vector-borne diseases as it may considerably affect transmission and the timing and intensity of outbreaks [2]. Thus, understanding vector feeding behavior and preferences, and the ecological factors underlying such preferences, is basic for a better knowledge of vector–pathogen–host relationships and their epidemiological consequences [3, 4]. Several factors should be considered when addressing such studies. First, such relationships are frequently complex since, multiple hosts and vectors are often involved [5–8]. Whereas it is generally accepted that pathogen dynamics are driven by the dynamics of the overall biological diversity of the community, the relationship between biological diversity and pathogen transmission is unclear [9–11]. For multihost/multivector models, it has been shown that local richness and composition of host reservoirs and vectors may lead to different combinatorial effects on disease transmission ([7] and references therein). Thus, as the former authors suggested, collection of qualitative information at local/regional scale, on the largest possible number of actors involved in disease dynamics, repeated for different periods of time, will help to infer regional disease dynamics and its effects on host assemblages (and vice versa). Additionally, the current “One Health” approach demands the study of wildlife host–vector relationships in order to understand and predict the hosts that are connected by different vectors [12].

Second, our knowledge of the role of wild hosts on many diseases is admittedly poor [13, 14] and information on vectors of most pathogens is scarce and frequently biased to specific pathogens, vector groups, and conditions [15]. This is the case of phlebotomine sandflies [16], which are vectors of the parasitic protozoan *Leishmania* spp. (Kinetoplastida: Trypanosomatidae), *Bartonella* bacteria, and phleboviruses [17–19]. Leishmaniasis is one of the most neglected disease, despite that fact that 350 million people are considered at risk of contracting it [20]. It has been endemic in all southern European countries for decades and its incidence in humans is relatively low, but underreporting and asymptomatic infection is common [21]. In Spain, for example, 22.1% of blood donors in Balearic Islands, a highly endemic area, tested positive by polymerase chain reaction (PCR) for *Leishmania* [22] and 8% tested positive in Murcia [23]. Therefore, sandflies constitute a major veterinary and public health concern [20]. Female sandflies are considered opportunistic blood feeders [24–28], but most studies on phlebotomine sandflies in Europe focus on human and mammals in close contact with humans (e.g., hares [14, 29] and references therein). Even though a crescent number of studies are exploring these relationships (i.e., for the neotropics [30, 31]), the role of wild vertebrates still remains uncertain despite some evidence of their involvement. For instance, Kocher et al. [32] exploring sites with different levels of human disturbance, found that while *Leishmania* prevalence decreases with mammal diversity, the latter is positively associated with sandflies density. This highlights the need for further research into the role of alternative blood sources, as they may sustain and shape vector populations and, via their population dynamics and ecology, affect the transmission cycles of pathogens [14, 33].

Third, the life-history traits, habitat and blood sources preferences of these vectors is mostly biased towards some specific species under laboratory conditions [19]. Most sandfly species are considered exophagic (i.e., feeding and egg development occur outdoors), but some species are known to be endophagic and endophilic [14, 16]. Some species have been shown to easily adapt to anthropic environments, including urban/suburban peridomestic habitats [26, 34, 35]. In rural areas, sandflies congregate in buildings housing domestic animals, but they are also found in less anthropized and particular habitats such as rabbit burrows, caves, and naturalized abandoned buildings, where they rely on wildlife for blood [36–38]. For instance, sandflies have been reported in nest boxes occupied by wild birds, with prevalence ranging 19%–27% [39]. Sandflies could use these enclosed habitats for resting and/or for feeding. Thus, ascertaining the endophagic preferences of sandfly species is important when evaluating the need for specific detection, surveillance, and prevention programs.

To address the knowledge gaps reported above, we undertook a local-scale study considering as many vector and blood source species as possible (including wild hosts [14]) likely involved in the dynamics of pathogens transmitted by sandflies in the vicinity of avian nests. For this, we studied the sandfly community in a rural, semiarid area, and focused on their feeding behavior and interaction with wildlife, particularly wild birds, as potential sources of blood. In contrast to the classic method of sampling in the vicinity of domestic animals (chicken coops, dog houses, and corrals), we focused our sampling effort close to habitat niches used by wild birds, namely, cavity-nesting birds breeding in natural holes in cliffs, seminatural openings (putlog holes in bridges), and nest boxes. Moreover, we combined untargeted trapping in the surroundings of such breeding sites with targeted trapping into the nests of one of the most abundant cavity-nesting bird species (the European roller *Coracias garrulus*). In this way, we aimed at addressing the following goals: (i) Identify the sandfly species in the vicinity of habitat niches used by wild birds in our study area, a semiarid region in southeastern Spain; (ii) ascertain their main blood sources and feeding behavior around such avian habitats; and (iii) describe the main characteristics of the trophic network constituted by this multihost/multivector community.

## 2. Material and Methods

### 2.1. Study Area and Study Species

**Figure 1:**
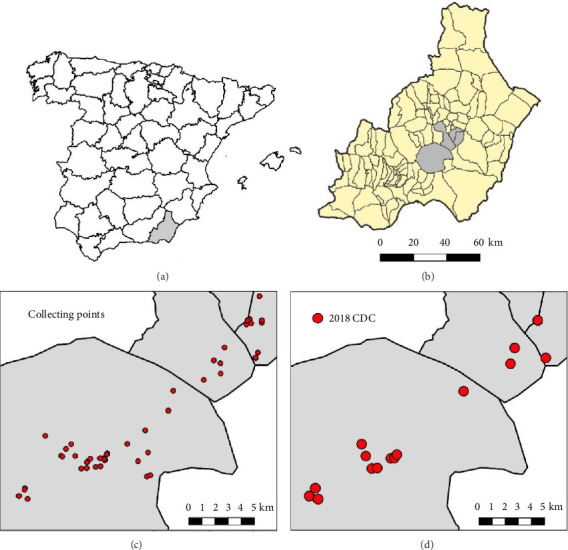
Study area: Almería province in Spain (A); studied municipalities in Almería province (B); localities where sandflies were collected during the study period, including CDC and sticky traps (C); localities where sandflies were sampled in 2018 using CDC traps (D). In the graphs, some nearby localities overlap. CDC, Centers for Disease Control.

The study area (~50 km^2^) is located in Campo de Tabernas (Almería, SE Spain, 37°05′N, 2°21′W), in the municipalities of Tabernas, Tahal, Uleila del Campo, and Benizalón (Figure 1). This is a subdesert mixed area with open shrubland and dispersed olive and almond plantations, dry riverbeds (*ramblas* in Spanish), and scattered inhabited and deserted farms. The climate is temperate, semiarid Mediterranean with a strong water deficit during hot summer months (June–September), when absolute maximum monthly temperature is higher than 40°C and the monthly average of the maximum daily temperatures remains above 30°C [40]. The average annual temperature is 18°C, with mild interannual oscillations of 3–4°C and significant intra-annual fluctuations [40]. The mean annual rainfall is ca. 230 mm with high interannual and intra-annual variability [41].

In this predominantly flat and treeless area, avian communities are more diverse in ramblas than in shrub-steppes, due to the availability of natural cavities in cliffs used by nonexcavator, cavity-nesting bird species (e.g., Common kestrel *Falco tinnunculus*, Eurasian hoopoe *Upupa epops*, Eurasian Jackdaw *Corvus monedula*, European roller *C. garrulus*, Little owl *Athene noctua*, Scops owl *Otus scops*, Rock pigeon *Columba livia*, etc.) [42]. In absence of ramblas, nonexcavator bird species aggregate in artificial structures such as deserted farmhouses and bridges [43]. In this area, the migratory European roller (roller hereafter) is one of the most common avian species, breeding from the end of April to mid-July in close contact with the previously described species but also with the Eurasian collared dove (*Streptopelia decaocto*), Common wood pigeon (*Columba palumbus*), Spotless starling (*Sturnus unicolor*), and House sparrow (*Passer domesticus*) when nesting on trees. Traditionally, rollers have bred in natural cavities located in cliffs and human constructions. Yet, due to a nest box installation program, currently rollers breed mainly in nest boxes [43, 44], whose location (trees, cliffs, or human constructions, e.g., bridges and farmhouses) has profound effects on their ectoparasite community, such as the higher prevalence of sandflies in nest on cliffs or farmhouses [39, 45]. Regarding mammals, wild boar (*Sus scrofa*), rabbit (*Oryctolagus cuniculus*), hare (*Lepus granatensis*), red fox (*Vulpes vulpes*), garden dormouse (*Eliomys quercinus*), domestic goat (*Capra aegagrus hircus*), and sheeps (*Ovis aries*) are frequently observed in the study area.

In addition to the nest box supplementation program, a natural cavity restoration scheme has also been performed to enhance breeding in natural and seminatural cavities [46]. This program consisted on reducing the entrance size of cavities in cliffs and bridges by overlaying a holed wooden plank. This technique allowed adhesive traps to be placed in the inner face of the wooden planks in order to trap sandflies and other vectors.

### 2.2. Sandfly Trapping

**Figure 2:**
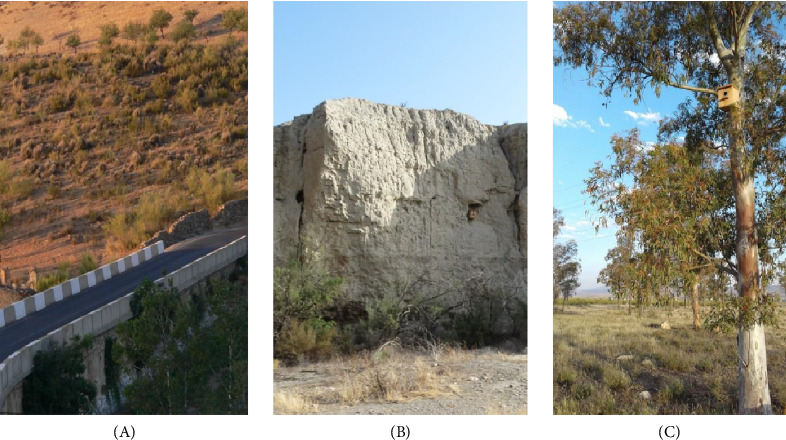
Habitat types sampled with CDC and sticky traps: bridges over ramblas (A); sandstone cliffs (B); isolated group of trees (C). CDC, Centers for Disease Control.

Sandflies were trapped during the roller breeding season using two methods: Centers for Disease Control (CDC) light traps and sticky traps (a total of 73 pairs of CDC traps and 2.48 m^2^ of sticky surface during the whole study period). CDC light traps were placed in three main habitat types where rollers and other cavity nesting birds breed: (i) sandstone cliffs, (ii) bridges over ramblas, and (iii) close to isolated trees or groups of trees with nest boxes. This approach provides information on sandfly species in lightly anthropised environments (sparse human population and lack of livestock farms) and in the most characteristic habitat types of the study area (Figure 2). In each sampling point two CDC traps (one with ultraviolet (UV) light and one with incandescent light) were put ca. 50 cm apart from each other and were also baited with carbon dioxide (CO_2_) to use as many different stimuli as possible. The catches from the two traps of each pair were pooled. Dry ice was used as source of CO_2_ (1 kg of dry ice per night per pair of traps to ensure the continued emission of CO_2_ until the collection of the traps at dawn). The traps were powered by a 6 V battery of 12 Ah. The trapping sessions were adjusted according to the breeding season of rollers and the lunar calendar, so that traps were active on the days during, or close to, the period of new moon to reduce the effect of ambient light [47]. Windy nights were also avoided. Trapping was performed during three breeding seasons: 2016, 2017, and 2018.

During 2016 and 2017, 20 sampling points along the study area were sampled once by CDC traps: eight points were located on cliffs, eight on trees, and four on bridges over ramblas. In 2016, one group of 10 pairs of traps were placed from 8 June to 10 June and a second group of 10 trap pairs were placed from 7 July to 8 July. In 2017, all 20 trap pairs were set from 22 June to 1 July. In 2018, four of the locations sampled in the previous years were not sampled and an additional one (in a farmhouse) was included. Thus, 17 sampling points were sampled by CDC traps (Figure 1): six points were located on cliffs, six close to trees, four on bridges, and one in a deserted farmhouse. Sixteen points were sampled twice, from 11 June to 14 June and from 10 July to 15 July, while the one in the farmhouse was sampled only once, on June 14. Most traps (89%) were set before dusk or shortly after and all were removed after sunrise. Captured insects were moved to the Estación Experimental de Zonas Áridas and frozen until identification.

Additionally, sticky traps were placed inside nest boxes and manipulated cavities occupied by rollers during the 2018 breeding season (from 18th June to 7th July). Specifically, sticky traps were fixed under the upper lid of 36 nest boxes (19 on trees, 12 on cliffs, and 5 on farmhouses). We followed the method described by Tomás et al. [48] (i.e., using Petri dishes smeared with body gel–oil as a nonattractant glue) but replacing Petri dishes by white vegetal papers (trap size = 330 cm^2^) that were fixed by thumbtacks on the inner side of the upper lid. Sticky traps were placed when the oldest nestling of each nest was approximately 13 days old. Each sticky trap was maintained for 4 days after which it was replaced by a new trap that was kept for a second period of 4 days. A similar procedure was used to sample three manipulated cavities on bridges occupied by rollers. In these cases, the traps were located in the inner surface of the wooden plank covering the entrance of the nest (trap size = 273, 342, and 441 cm^2^) and were maintained 4, 6, and 7 days, respectively. The differences between the traps sizes were due to cavity shape. Collected insects were kept in 70% ethanol and frozen.

### 2.3. Identification of Insects and Blood Meal Sources

The composition of the sandfly community was studied with data from CDC traps during 2018, the year with the highest sampling effort. Most nonengorged phlebotomines captured were mounted on slides and morphologically identified according to the usual structures (cibarium, pharynx, and spermathecae in females and genitalia in males) following Gállego-Berenguer et al. [49]. Additionally, engorged individuals from CDC traps from all 3 years, and from sticky traps from 2018, were identified using molecular methods.

To identify blood meal sources of captured phlebotomines, we used DNA barcoding to analyze individually 95 visually engorged females captured using either method (sticky traps from 2018 and CDC traps from 2016 to 2018). Because nonvisually engorged phlebotomines can include traces of undigested blood [50] we also analyzed 79 nonengorged specimens (64 gravid females and 15 nongravid females) captured by CDC traps from 2018. In addition to identifying the blood meal source, DNA barcoding was also used to identify the sandfly species.

Identification of the engorged sandflies was established by Sanger sequencing of the barcode region of cytochrome c oxidase subunit I (COI) following Hernández-Triana et al. [51]. Briefly, the barcode region was amplified via PCR using the primer cocktail C_LepFolF + C_LepFolR. The amplicons were diluted fourfold with sterile water and sequenced on the DNA analyzer ABI 3730XL (Applied Biosystems) using BigDye chemistry. The resulting traces were edited using CodonCode (CodonCode Corporation, Ded-ham, Massachusetts) and uploaded to the Barcode of Life Database (BOLD). Full details for each specimen and sequence information can be found at the BOLD within the “Human Pathogens and Zoonoses Initiative, Working Group 1.4, Project XENAL-Xenosurveillance and DNA barcoding bloodfed sandflies from Tabernas Desert, Almeria, Spain (SANFA, SANFL)”. The digital object identifier (DOI) for the publicly available dataset project in BOLD is dx.doi.org/number requested/DS-SANDFSP. Accession numbers for all sequences were obtained from NCBI (accession numbers: OR076126–OR076314). Comparison to the comprehensive BOLD reference library was used to assign an identification to each specimen.

Identification of the vertebrate blood source of engorged female sandflies was established by high-throughput sequencing (HTS) following Estrada-Franco et al. [52], with minor modifications. HTS allows the identification of different DNA fragments from the same sample simultaneously. Briefly, the same DNA extract used to identify the sandfly specimens was used to identify residual DNA leftover from the blood meal. Due to the degraded nature of the blood DNA, a small fragment of the DNA barcode region (185 bp) was amplified via PCR using the primer cocktails C_BloodmealF1_t1 + Mod.Mam.R_T1 ([53]; modified primer C_VR1di_t1 in [54]). The resulting amplicons were all tailed with M13 sequences, which served as a universal priming site for PCR-indexing. The amplicons from each specimen were indexed with a unique molecular identifier (UMI) from the “IonCode” set (Thermo Scientific). The indexed amplicons were then pooled, purified, and sequenced on an Ion Torrent Personal Genome Machine (PGM) (Thermo Scientific) at 22 pM loading concentration. The raw sequence reads were filtered for length and quality, trimmed of primer and adapter sequences, clustered into operational taxonomic units (OTUs), and identified by comparing to a vertebrate COI reference library, assigning species identification when sequence similarity was over 98% (for bioinformatic details, see [52]). Since wild boar has identical sequences to their domestic counterpart, we decided to assign the blood meals to the wild species based on the widespread presence of wild populations and the scarcity of pig livestock in the region. To exclude possible contamination (exogenous or cross-contamination between samples), two strategies were employed: (i) reads in negative control wells were proportionally subtracted from all other samples and (ii) OTUs were only accepted as genuine if they were composed of at least 100 raw reads. These measures act to mitigate instances of false positive detections.

### 2.4. Statistical Analysis

Individuals captured with CDC traps during 2018, and in which the blood meal was identified, were used to quantitatively analyze the bipartite interaction network between the phlebotomine and the vertebrate blood source based on the graph theory. Several coefficients were used: (i) Degree of specialization (H_2_′): it is a network-level index which characterizes the degree of specialization in the entire network and can be used for comparisons across different interaction webs (range: 0–1; H_2_′ = 0 in case of no specialization, where all species interact with each other equally [55]); (ii) nestedness (nestedness metric based on overlap and decreasing fill (NODF)): metric based on overlap and decreasing fill for binary bipartite networks which measures the extent to which the interactions of less connected species are subsets of the interactions of more connected ones (range: 0–100; NODF = 0 no nestedness, i.e, the interactions do not show any nested pattern [56]); (iii) degree of interspecific competition by means of *C*-score which, in host–parasite network, quantifies how often pairs of parasites are found on the same host compared to how often are found on different hosts (range: 0–1, with values close to 0 indicating aggregation and values close to 1 indicating competition [57]); (iv) connectance (C): the ratio links/species^2^ (empirical range: 0.02–0.4 [58]), which measures the proportions of realized interactions in relation with all the possible interactions [59]; (v) dependence: quantifies the imbalance between the interaction strengths of a species pair showing the dependence relationships between pair members, that is, how reliant a parasite is on a particular host (range: 0–1, with values close to 0 indicating a generalized pattern, where the parasite or the host does not depend heavily on a single interaction [60]); (vi) betweenness centrality (based on Dijkstra's algorithm): measures the number of times one node serves as a bridge within the shortest path that connects two other nodes, which in an ecological network means the number of relations that this node (taxon) supports and, hence, how critical is the role of this node in maintaining the structure of the network (range: 0–1, with values close to 0 occurring in nodes with less critical role for network structure [61]); and (vii) modularity (based on QuanBiMo algorithm, [62]): the organization of the network into modules, which corresponds to a tightly interconnected set of edges in a network [63] (range from 0 when there is no more links within modules than expected by chance to 1 for networks composed of perfect modules). Modularity *Q* depends on network size [64] and, thus, the absolute value was corrected using null models based on the random placement of interactions observing the same marginal totals [65]. Corrected *Q* was calculated as the difference between the value of the empirical network and the mean value obtained from 1000 null models for QuanBiMo [66].

A *χ*^2^ test was used to explore the association between the number of visually engorged females of each species and the method of capture (data from 2018) and a Fisher test was used to test the feeding preferences of the most common phlebotomine sandfly species captured by CDC traps in 2018 for mammalian or avian source of blood.

Analyses were conducted with RStudio version 1.4.1106 [67] under R language [68] and the packages ade4, adespatial, bipartite, FactoMineR, factoextra, igraph, and vegan [69–75].

## 3. Results

### 3.1. Sandfly Community

In total, 3532 phlebotomines were captured by CDC traps during the three study years. During 2016, 752 phlebotomines were collected (mean abundance per pair of traps and standard error (SE) = 37.6 ± 12.4, *Q*1 = 3.7, *Q*3 = 37.5, and *N* = 20 pair of traps from 20 different sampling sites) and a similar sampling effort in 2017 resulted in a similar number of captures: 808 phlebotomines (mean abundance per pair of traps and SE = 40.4 ± 10.6, *Q*1 = 14.2, *Q*3 = 45.2, and *N* = 20 pair of traps from 20 different sampling sites). During 2018, when the sampling effort with CDC traps increased by sampling each site at two different dates, 1972 phlebotomines were collected (mean abundance per pair of traps and SE = 59.7 ± 10, *Q*1 = 12, *Q*3 = 97, and *N* = 33 samplings from 17 sites). Out of these sandflies, 841 were males, 1127 females, and 4 unsexed (Table 1). All but one of the pairs of CDC traps set in 2018 (at a tree sampled in July) caught phlebotomine sandflies.

Six species of phlebotomines were detected by means of CDC traps in 2018 (when all the individuals were subject to identification): *P. alexandri*, *P. ariasi*, *P. papatasi*, *P. perniciosus*, *P. sergenti*, and *Sergentomyia minuta*, being *P. perniciosus* the most abundant species, followed by *P. papatasi*, and *S. minuta* (Table 1). During 2016 and 2017, when only the engorged individuals were subject to identification, *P. ariasi*, *P. papatasi*, *P. perniciosus*, and *S. minuta* were found (Table 1).

Regarding catches in sticky traps placed in roller nests in 2018, we obtained 200 phlebotomines (78 from 3 bridge cavities and 122 from 36 nest boxes; prevalence = 0.46; confidence interval 95% = 0.30–0.63; mean abundance and SE = 5.13 ± 1.59, and *N* = 39 nests). Engorged individuals trapped in the nest boxes (*N* = 29) represented only a fraction of the sandfly community in the area, since only three species (*P. papatasi*, *P. perniciosus*, and *P. sergenti*) were found.

### 3.2. Blood Sources and Feeding Pattern of Sandflies

A total of 174 females were molecularly analyzed to identify the blood source, namely, 95 blood-fed, 64 gravid, and 15 nongravid females. Blood meal sources were successfully identified in 118 sandflies (67.81%), although 136 blood meal sources were identified due to 15 (12.7%) sandflies having mixed blood meals (12 double and 3 triple). The blood meal source was identified successfully in 75.8% (72 out of 95) of the visually engorged females, 57.6% (38 out of 64) of the gravid females, and 53.3% (8 out of 15) of the nongravid females.

Thirteen species of vertebrates were identified as blood sources of phlebotomines captured by means of CDC traps (Table 2). We recorded seven mammals: cow *Bos primigenius taurus* (L. 1758), domestic goat *Capra aegagrus hircus* (L. 1758), human *Homo sapiens* (L. 1758), hare *L. granatensis* (Rosenhauer 1856), rabbit *O. cuniculus* (L. 1758), sheep *O. aries* (L. 1758), and wild boar *S. scrofa* (L. 1758). Additionally, we identified six bird species: Graylag goose *Anser anser* (L. 1758), Rock pigeon *C. livia* (Gmelin 1789), European roller *C. garrulus* (L. 1758), Eurasian jackdaw *C. monedula* (L. 1758), turkey *Meleagris gallopavo* (L. 1758), and Eurasian hoopoe *U. epops* (L. 1758). The most frequent identification was human (73 cases), which was found mainly in *P. perniciosus* (34 cases) and *P. papatasi* (15 cases).

Blood meals of phlebotomine captured in sticky traps placed into roller nest cavities revealed the contact with four species of vertebrates (Table 2): two mammals (rabbits and humans) and two birds (rollers and pigeons). The most frequent identification was roller (27 cases), which was found in *P. papatasi* (20 cases), *P. sergenti* (six cases), and *P. perniciosus* (one case; Table 2).

Among the blood-fed individuals, 66 were captured by CDC traps (40 in 2018, 18 in 2017, and 8 in 2016), and 29 by sticky traps in 2018. Data from 2018 revealed that 3.55% (40 out of 1127) of the females trapped by CDC were visually engorged, namely, 4 *P. alexandri* (9.09% of 44 females), 1 *P. ariasi* (50% of 2 females), 14 *P. papatasi* (5.79% of 242 females), 17 *P. perniciosus* (2.44% of 698), 1 *P. sergenti* (20% of 5 females), and 2 *S. minuta* (1.49% of 134 females). Additionally, one engorged individual could not be identified. The engorged species in nest boxes were *P. papatasi* (*N* = 20), *P. perniciosus* (*N* = 2), and *P. sergenti* (*N* = 7). We found a significant association between the method of capture and the number of engorged individuals of the three species found in both types of traps (*χ*^2^ = 17.3; degrees of freedom (df) = 2; *p*-value < 0.001). Particularly, *P. papatasi* and *P. sergenti* were captured more frequently by sticky traps inside nest boxes, while *P. perniciosus* was captured more frequently by CDC traps. Engorged individuals of *P. perniciosus* were hardly captured in the nests and no *S. minuta* was trapped there. In contrast, *P. papatasi* was seemingly a common visitor of nest boxes and the occurrence of *P. sergenti* in nest boxes was much higher than expected (seven engorged individuals were found in nests, whereas only one engorged female was captured by means of CDC traps; Table 1).

### 3.3. Trophic Network

**Figure 3:**
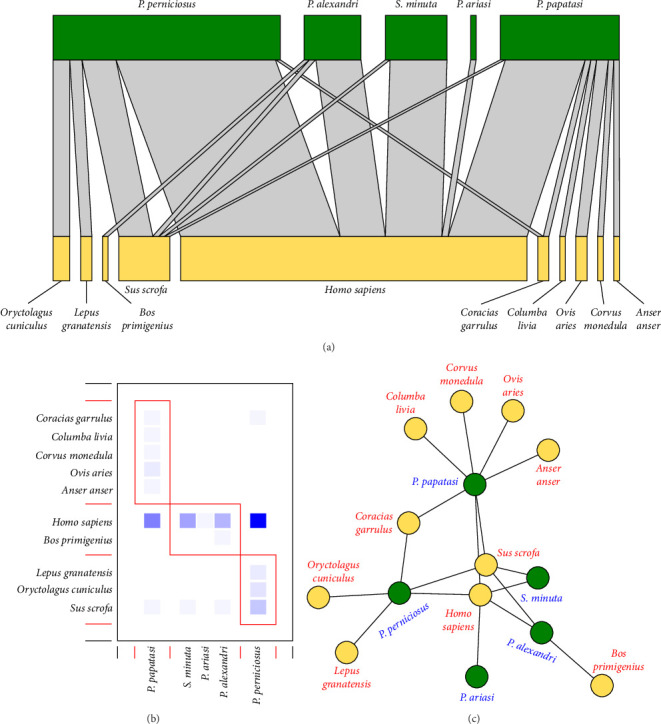
Network of hosts and sandflies from identified blood meals of individuals captured in 2018 by CDC traps. (A) Quantitative bipartite interaction network between the vertebrate hosts and the phlebotomine parasites. (B) The adjacency matrix of blood meals network, organized into three modules. Colored cells represent the gradient of interactions: from white (lower number of interactions) to deep blue (higher number of interactions). (C) Unipartited network, which links mean shared sandfly species (green dots) among host species (yellow dots). CDC, Centers for Disease Control.

Blood-fed females captured by CDC traps in 2018 were used to study the relationship between vectors and their blood sources (*N* = 83) by quantitative bipartite interaction network (Figure 3). The network is evidently poorly specialized (H_2_′ = 0.12), moderately nested (NODF = 58.06), with low interspecific competition between sandflies species (*C*-score = 0.0971; i.e., aggregation) since vector hosts share many of them (*C*-score = 0.42), and with high connectance (*C* = 0.36). The adjacency matrix of the blood meal network is organized in three modules (Figure 3B) with the interactions tending to occur similarly within and between subsystem (modularity *Q* = 0.15; *Q* corrected = 0.02). This network reveals that *P. papatasi* is the most opportunistic blood feeder. Its highest value of dependence is for *H. sapiens* (0.67) but this species also gathers the majority of bird blood sources (with low dependence values, <0.1). The access to both birds and mammals places *P. papatasi* as the main node of the network (betweenness centrality = 0.54). *Phlebotomus perniciosus* has even higher dependence for *H. sapiens* (0.91) and ranks second in the network (betweenness centrality = 0.31). Concerning blood sources, only *H. sapiens* (betweenness centrality = 0.36), *S. scrofa* (betweenness centrality = 0.21), and *C. garrulus* (betweenness centrality = 0.05) have a key role in the net being highly connected with different sandflies species (Figure 3C).

A comparison of blood source preferences of the two most abundant species, *P. papatasi* and *P. perniciosus*, reveals that they fed mainly on mammals, with *H. sapiens* being the main blood source for both species. Yet, there is a significant association between both sandfly species and the vertebrate group (mammals/birds) in which they fed (Fisher test: *F* = 0.04; *p*  < 0.05), with *P. papatasi* taking more bird blood meals (19.05%) than *P. perniciosus* (2.5%).

## 4. Discussion

Feeding behavior of blood-sucking insects plays a central role in the transmission and maintenance of vector-borne pathogens [76]. However, our knowledge of the feeding behavior of phlebotomines is biased towards urban and periurban areas (i.e., [77–80]; but see [32]), neglecting wildlife species and nonmammal hosts as blood sources [14] even though they may play an important role in the epidemiology of vector borne diseases [33, 81]. Here, we provide information on the trophic interactions between the community of sandflies close to avian nests and their blood sources in a semiarid area with scattered human settlements and evaluate the importance of wild species for these blood-feeding insects.

Previous work in the area, mainly based on sticky traps, reported diverse results in terms of species composition. Ortega [82] caught only *P. papatasi* and *P. perniciosus*. Sanchís-Marín, Villegas, and Morillas-Márquez [83] recorded the same six species captured in this study in Uleila del Campo, but only five in Tabernas (no *P. ariasi*). Gonzálvez et al. [84] carried out a specific study in a zoo in the area, where they collected *P. papatasi*, *P. perniciosus*, *P. ariasi*, and *S. minuta*. Some *P. perniciosus* were identified as *Phlebotomus* (*Larrousius*) *longicuspis* in these studies, but the presence of this species in Spain has been rejected and it is assumed that all records of *P. longicuspis* belong to *P. perniciosus* [85, 86]. Our study, including two sampling methods, a higher sampling effort, and specific sampling in various habitat types, resulted in the capture of higher numbers of sandflies and detection of six different species: *P. alexandri*, *P. ariasi*, *P. papatasi*, *P. perniciosus*, *P. sergenti*, and *S. minuta*. Interestingly, we recorded *P. alexandri* in several caches, a rare species which has been cited a few times before in the southeast Spain [87, 88]. Nevertheless, recently Bravo-Barriga et al. [89] did not record this species, despite a greater sampling effort with the same sampling trap at sites near to ours. As they suggested, the location of the traps (close to ruminant farms) could explain the absence of *P. alexandri*. In parallel, our sampling scheme, focusing on avian nests, may have biased the species of sandflies found, as well as the species providing blood meals. Although our study covers 3 years, sampling always took place during the dry months, which could affect the abundance and composition of the sandfly community and its feeding behavior. Further studies covering the whole period of sandfly activity could provide additional details on their composition and dynamics.

We found that *P. perniciosus* and *P. papatasi* were the most abundant species in the CDC traps (63.5% and 17.9%, respectively). Both species are commonly found in arid and semi-arid areas of the region [37, 82], with the presence of *P. perniciosus* being positively associated with rural environments far from villages [90] and areas with scrub vegetation and olive groves [91], which aligns with the characteristic of our study area. The dominance of *P. perniciosus* and *P. papatasi* agrees with the findings of Sanchís-Marín et al. [92] in the same province using CDC traps. In contrast, Sanchís-Marín, Villegas, and Morillas-Márquez [83] and Sanchís-Marín et al. [92] found that *S. minuta* was the most abundant species when using sticky traps, probably due to its behavior (less phototropic and frequenting crevices [49]). Furthermore, our results show that the number of engorged individuals of some species is associated with the method of capture. Therefore, a combination of different sampling methods targeting different sources of blood is essential to reveal the structure and composition of the whole sandfly community.

### 4.1. Blood Sources and Feeding Pattern of Sandflies

Our sampling design allowed us to identify the main blood sources of sandflies near avian nests and highlighted the importance of considering wild species in addition to domesticated animals. Six phlebotomines species fed on 13 vertebrate species, including mammals (seven species) and birds (six species). Overall, even though we sampled far from human settlements, humans were the most common blood source of phlebotomines, followed by rollers and wild boar. Human ubiquity (there are disseminated houses/farms in the study area) together with the mobility detected for some sandflies, could account for this result. Although sandflies are not considered good flyers [19], some authors reported movements over 1.0 km [93–96] that could be associated with vertebrate presence, wind speed, and wind direction [94]. All other identified mammals have been previously recorded as sandfly hosts in the Iberian Peninsula [24, 25, 27, 28, 50, 96–102]. However, there are very few records of wild avian sources of blood of sandflies, and most studies are focused on domestic poultry [26, 102, 103].

Although sandflies are known to feed on wild animals [14, 27, 81], the number of studies on the importance of wildlife in their diet is limited [29, 104–106], but growing recently. Kocher et al. [32] found evidence of increased phlebotomine sandflies under scenarios of increased mammalian diversity and highlighted the importance of this variable in *Leishmania* infection, and Barbero-Moyano et al. [107] analyzing wild lagomorphs of Andalusia found 56.4% of them exposed to *L. infantum*. Our results show that wild animals, and in particular cavity-breeding wild birds, may be an important source of blood for sandflies near bird nests. Namely, out of the 13 identified species, three were wild mammals (rabbit, wild board, and hare) and four were wild birds (roller, pigeon, jackdaw, and hoopoe), representing 15.4% and 24.3% of the total of blood meals, respectively. At least five phlebotomine (*P. ariasi*, *P. papatasi*, *P. perniciosus*, *P. alexandri*, and *S. minuta*) fed on wild mammals and three species (*P. papatasi*, *P. perniciosus*, and *P. sergenti*) fed on wild birds. *P. papatasi* is responsible for the majority of avian feeding, while *P. sergenti* is the most exclusive avian blood feeder, results that are supported by Svobodová et al. [103]. Birds as blood source could be especially important in habitats such as ramblas, and around human constructions, where the abundance of sandflies is high. In contrast, the opposite is true in woody patches [39]. Under this scenario, wild animals may contribute to the maintenance of sandfly populations, and some species may act as reservoirs of pathogens transmitted by sandflies (*Leishmania* and Phleboviruses). However, identification of reservoirs would require confirmation of pathogen persistence and potential to transmit it [108], as well as ecological and parasitological analyses to determine whether these potential reservoirs can serve as an actual reservoir in a given environment [105].

Most sandflies fed from mammals were found in CDC traps. Two wild bird species as sandfly blood sources were found only in CDC traps, while other two wild bird species were found in sandflies trapped in both CDC and sticky traps. Since the attracting power of different types of traps and the efficacy of various types of attractants vary among sandfly species [109], it is recommended to combine several trapping methods in different locations to obtain real information on this complex network of intereactions.

The importance of the roller as a blood source found in this study is obviously due to specific sampling in rollers nests, since there is a high probability of obtaining blood meals from the animals occupying the dwellings where traps are set [110]. Most of the blood meals obtained from rollers nests are from rollers (27 out of 31 individuals from three species: *P. perniciosus*, *P. papatasi*, and *P. sergenti*). *Phlebotomus papatasi* and *P. sergenti* are considered endophilic, while *P. perniciosus* is considered exophilic [19, 111], although it has been found inside cavities [38]. Our results on the feeding behavior of these species agree with previous reports, although they suggest that *P. perniciousus* has a certain capacity to feed in closed habitats. Interestingly, individuals of *P. papatasi* and *P. perniciosus* captured in rollers nests also had blood meals from other species (human, rabbit, and pigeon), suggesting the use of enclosed habitats also for resting.

We obtained valuable information of host-use, analyzing not only visually engorged sandflies but also gravid and nongravid females. Approximately 50% of these individuals provided evidence about the blood source, thus contributing to get a more complete view of the host–vector relationship. Trace DNA studies are common in studies of host use by ticks, where several months may pass between blood ingestion and capture [112]. Further studies of host DNA traces in nonengorged individuals [50] could be useful to increase knowledge on the feeding behavior of sandflies. However, it is necessary to know the factors that may bias these results. For example, Léger et al. [113], working with ticks, found that mammal blood is generally easier to detect than that of birds.

### 4.2. Trophic Network

Understanding pathogen dynamics and the transmission of infectious diseases requires a network perspective that considers that multiple hosts and vectors are frequently involved [5, 6, 8, 114]. Several authors have suggested that applying such perspective at a local or regional scale would greatly improve our knowledge on disease transmission and dynamics [7, 114]. Specifically, Roche and Guégan [7] suggested that more attention should be paid to recording the number and identity of all potential vector and reservoir species locally, instead of focusing only on the more competent, well-accepted blood source and vectors. Following this approach, but admitting limitations in the coverage of the different potential host species due to the sampling design (three habitat types), we have highlighted the main features of the interaction networks between a community of six phlebotomine sandflies detected near avian nests and their domestic and wild blood sources in a semiarid environment, namely, interactions between vertebrates and sandflies in our scenario evidence a generalized network where sandflies species are linked with multiple vertebrete species and share most of them in a highly connected network. Sandflies species aggregate within the blood source species and show low levels of competition. Therefore, the results of the network analysis match with the opportunist behavior described for sandflies [19, 28], where parasite species feed on multiple species depending mostly on occurrence and not on feeding preference. The moderately nested network reveals that sandflies with few connections are a subset of the links of the most connected ones. This means that specialized sandflies interact with vertebrates that attract many sandflies species, and generalist sandflies interact with these vertebrates as well as with those that attract fewer sandflies (Figure 3A) [115]. These patterns agree with those obtained for other ectoparasites [116] and with the general rule for ectoparasite network nestedness obtained by Graham et al. [115] for different ectoparasite–vertebrate hosts networks.

Apart from humans, we could identify 12 other species contributing to the maintenance of sandflies populations, with wild boar being the most important nonhuman blood source. Other vertebrate could also play an important role from the perspective of pathogen transmission. Wild hares and rabbits have been proved effective transmitters of *L. infantum* to *P. perniciosus* [29, 117, 118]. A study near to our study area (approximately 150 km) showed high rates of infection and heavy parasite burdens in apparently asymptomatic rabbits and also in *P. perniciosus* females [119]. We found wild hares and rabbits as blood source of *P. ariasi*, *P. perniciosus*, and *P. papatasi*. *Phlebotomus ariasi* and *P. perniciosus* are the main vectors of *L. infantum* in Western Europe and North Africa, and *P. papatasi* is the main vector of *L. major* in North Africa and the Middle East [120].

We also found that at least four of the sandfly species fed on humans together with other species, thus potentially bringing in contact parasites from different species. For example, *P. papatasi* and *P. perniciosus* are the most connected species in the network (Figure 3), and both species together are able to fed on almost all of the species (except cows), connecting the entire network. The generalist nature of *P. papatasi* gives it a main role in this network, given its relationship with both mammals (mainly humans) and birds. It is the species with the broadest blood source range and the most ornithophilic. *Phlebotomus perniciosus* has the second highest number of relationships (but seldom with birds). Both species deserve further attention, as they share several blood sources, are effective vectors of *L. major* and *L. infantum*, and present the highest values of centrality in the network. That being said, other sandfly species could also be important transmitters of the disease. For example, *S. minuta* is suspected to be involved in the cycle of mammal vectorial diseases, since *L. major* and *L. infantum* [121–124] and Toscana virus [125] have been found in this sandfly.

Leishmaniasis in Andalucia is a notifiable disease. Almeria province is an endemic area with intermediate-high risk of canine *L. infantum* infection [126]. Sanchís-Marín et al. [92] reported a prevalence of canine leishmaniasis of 4.5% for the province and 11.5% for Tabernas. Concerning human leishmaniasis, clinical data from 2003 to 2020 report only 14 cases from Almería and none from our study area (Junta de Andalucía, personal communication). It is tempting to explain this data based on the effect of biological diversity on disease dynamics. Host and vector richness and composition are known to influence pathogen prevalence and, thus, disease transmission [5, 6, 8]. Studies indicate that local communities with low richness in reservoir host species tend to present higher levels of disease prevalence, whereas local communities that involve a larger number of reservoir hosts species see this level decreasing [7]. Our study found 13 species acting as blood sources (including humans), which is probably an underestimate. However, there are additional explanations for the apparently low prevalence of leishmaniasis in our study area: (i) this infection can pass unnoticed or can be misdiagnosed and (ii) dogs, the main reservoir of *Leishmania* parasites, may be rare in the area. Indeed, no blood meals from dogs were found. Alternatively, it could be that sandflies in our area prefer other species. In any case, estimating the risk of leishmaniasis requires considering the role of wild and domestic species as pathogen host, their relative abundance, their competence for pathogen amplification, and vector host preference [3].

## 5. Conclusions

Our study investigates the feeding behavior of sandflies near bird nests in a semiarid habitat. Although our sampling design might have biased the phlebotomine sandfly species and vertebrates found in blood meals, the results contribute to identify the host use range of sandflies, with special emphasis on the role of wild species, and to complete our knowledge of sandfly interactions. For the latter, we apply the suggested network perspective of sampling biting insects and their respective blood sources at the local scale [114]. This allows us to identify the most important phlebotomine sandfly species and blood sources near avian habitats, to understand the role of each species in maintaining sandflies, and to identify opportunities for coinfection and pathogen transmission by sandflies. This study provides baseline data on poorly studied pathogen–vector–host pathways for the transmission of *Leishmania*.

## Figures and Tables

**Table 1:** Phlebotomines identified molecularly or taxonomically and its classification according to year, trapping method, species, and sex.

Year	Capture	Genus	Species	Male	Female	Unsexed	Molecularly analyzed
2016	CDC	*Phlebotomus*	*P. ariasi*	0	1	0	1
	“	“	*P. papatasi*	0	1	0	1
	“	“	*P. perniciosus*	0	4	0	4
	“	*Sergentomyia*	*S. minuta*	0	2	0	2

2017	CDC	*Phlebotomus*	*P. ariasi*	0	1	0	1
	“	“	*P. papatasi*	0	8	0	8
	“	“	*P. perniciosus*	0	8	0	8
	“	*Sergentomyia*	*S. minuta*	0	1	0	1

2018	CDC	*Phlebotomus*	*P. alexandri*	32	44	0	14
	“	“	*P. ariasi*	2	2	0	1
	“	“	*P. papatasi*	111	242	0	29
	“	“	*P. perniciosus*	552	698	0	50
	“	“	*P. sergenti*	5	5	0	1
	“	“	Unidentified	2	2	4	1
	“	*Sergentomyia*	*S. minuta*	137	134	0	23
	Sticky	*Phlebotomus*	*P. papatasi*	0	20	0	20
	“	“	*P. perniciosus*	0	2	0	2
	“	“	*P. sergenti*	0	7	0	7

Total	—	—	—	841	1182	4	174

*Note:* In 2016 and 2017, only blood-fed individuals were identified. All individuals trapped in 2018 with CDC traps were identified, whereas for sandflies captured in 2018 with sticky traps, only blood-fed individuals were identified. The symbol (“) stands for the same as previously stated.

Abbreviation: CDC, Centers for Disease Control.

**Table 2:** Host species identified as blood source for female sandflies in south-eastern Spain during three years.

A					B			
Year	Sandfly species	Host	Trap	*N*	Host	Sandfly species	Trap	*N*
2016	*Phlebotomus ariasi*	*Oryctolagus cuniculus*	CDC	1	*Homo sapiens*	*Phlebotomus perniciosus*	CDC	3
	*Phlebotomus perniciosus*	*Homo sapiens*	CDC	3	*“*	*Sergentomyia minuta*	CDC	2
	*Sergentomyia minuta*	*Homo sapiens*	CDC	2	*Oryctolagus cuniculus*	*Phlebotomus ariasi*	CDC	1

2017	*Phlebotomus ariasi*	*Homo sapiens*	CDC	1	*Capra aegagrus hircus*	*Phlebotomus papatasi*	CDC	1
	*Phlebotomus papatasi*	*Capra aegagrus hircus*	CDC	1	*Homo sapiens*	*Phlebotomus ariasi*	CDC	1
	*“*	*Homo sapiens*	CDC	1	*“*	*Phlebotomus papatasi*	CDC	1
	*“*	*Meleagris gallopavo*	CDC	1	*“*	*Phlebotomus perniciosus*	CDC	3
	*“*	*Oryctolagus cuniculus*	CDC	1	*“*	*Sergentomyia minuta*	CDC	1
	*“*	*Sus scrofa*	CDC	2	*Meleagris gallopavo*	*Phlebotomus papatasi*	CDC	1
	*Phlebotomus perniciosus*	*Homo sapiens*	CDC	3	*“*	*Phlebotomus perniciosus*	CDC	1
	*“*	*Meleagris gallopavo*	CDC	1	*Oryctolagus cuniculus*	*Phlebotomus papatasi*	CDC	1
	*“*	*Sus scrofa*	CDC	2	*Sus scrofa*	*Phlebotomus papatasi*	CDC	2
	*Sergentomyia minuta*	*Homo sapiens*	CDC	1	*“*	*Phlebotomus perniciosus*	CDC	2

2018	*Phlebotomus alexandri*	*Bos primigenius taurus*	CDC	1	*Anser anser*	*Phlebotomus papatasi*	CDC	1
	*“*	*Homo sapiens*	CDC	8	*Bos primigenius taurus*	*Phlebotomus alexandri*	CDC	1
	*“*	*Sus scrofa*	CDC	1	*Columba livia*	*Phlebotomus papatasi*	CDC	1
	*Phlebotomus ariasi*	*Homo sapiens*	CDC	1	*“*	*“*	Sticky	1
	*Phlebotomus papatasi*	*Anser anser*	CDC	1	*Coracias garrulus*	*Phlebotomus papatasi*	CDC	1
	*“*	*Columba livia*	CDC	1	*“*	*“*	Sticky	20
	*“*	*“*	Sticky	1	*“*	*Phlebotomus perniciosus*	CDC	1
	*“*	*Coracias garrulus*	CDC	1	*“*	*“*	Sticky	1
	*“*	*“*	Sticky	20	*“*	*Phlebotomus sergenti*	Sticky	6
	*“*	*Corvus monedula*	CDC	1	*Corvus monedula*	*Phlebotomus papatasi*	CDC	1
	*“*	*Homo sapiens*	CDC	14	*Homo sapiens*	*Phlebotomus alexandri*	CDC	8
	*“*	*“*	Sticky	1	*“*	*Phlebotomus ariasi*	CDC	1
	*“*	*Oryctolagus cuniculus*	Sticky	1	*“*	*Phlebotomus papatasi*	CDC	14
	*“*	*Ovis aries*	CDC	2	*“*	*“*	Sticky	1
	*“*	*Sus scrofa*	CDC	1	*“*	*Phlebotomus perniciosus*	CDC	28
	*Phlebotomus perniciosus*	*Coracias garrulus*	CDC	1	*“*	*“*	Sticky	1
	*“*	*“*	Sticky	1	*“*	*Phlebotomus sp*.	CDC	1
	*“*	*Homo sapiens*	CDC	28	*“*	*Sergentomyia minuta*	CDC	10
	*“*	*“*	Sticky	1	*Lepus granatensis*	*Phlebotomus perniciosus*	CDC	2
	*“*	*Lepus granatensis*	CDC	2	*Oryctolagus cuniculus*	*Phlebotomus papatasi*	Sticky	1
	*“*	*Oryctolagus cuniculus*	CDC	3	*“*	*Phlebotomus perniciosus*	CDC	3
	*“*	*Sus scrofa*	CDC	6	*Ovis aries*	*Phlebotomus papatasi*	CDC	2
	*Phlebotomus sergenti*	*Coracias garrulus*	Sticky	6	*Sus scrofa*	*Phlebotomus alexandri*	CDC	1
	*Phlebotomus* sp.	*Homo sapiens*	CDC	1	*“*	*Phlebotomus papatasi*	CDC	1
	*“*	*Upupa epops*	CDC	1	*“*	*Phlebotomus perniciosus*	CDC	6
	*Sergentomyia minuta*	*Homo sapiens*	CDC	10	*“*	*Sergentomyia minuta*	CDC	1
	*“*	*Sus scrofa*	CDC	1	*Upupa epops*	*Phlebotomus* sp.	CDC	1

*Note*: Information is sorted by year, sandfly species, and host species (A) and by year, host, and sandfly species (B). Information about the type of trap and the number of blood meals from each host and vector species is also shown. The symbol (“) stands for the same as previously stated.

Abbreviation: CDC, Centers for Disease Control.

## Data Availability

The data that support the findings of this study are available from the corresponding author, J. V., upon reasonable request.
